# miR-141 is involved in BRD7-mediated cell proliferation and tumor formation through suppression of the PTEN/AKT pathway in nasopharyngeal carcinoma

**DOI:** 10.1038/cddis.2016.64

**Published:** 2016-03-24

**Authors:** Y Liu, R Zhao, H Wang, Y Luo, X Wang, W Niu, Y Zhou, Q Wen, S Fan, X Li, W Xiong, J Ma, X Li, M Tan, G Li, M Zhou

**Affiliations:** 1 Hunan Cancer Hospital and The Affiliated Tumor Hospital of Xiangya School of Medicine, Central South University, Changsha, Hunan 410013, PR China; 2 Key Laboratory of Carcinogenesis of Ministry of Health and Key Laboratory of Carcinogenesis and Cancer Invasion of Ministry of Education, Cancer Research Institute, Central South University, Changsha, Hunan 410078, PR China; 3 Department of Pathology, The Second Xiangya Hospital, Central South University, Changsha, Hunan 410011, PR China; 4 Department of Gastroenterology, The Third Xiangya Hospital, Central South University, Changsha, Hunan 410013, PR China; 5 Mitchell Cancer Institute, University of South Alabama, Mobile, AL 36604, USA

## Abstract

Bromodomain containing 7 (BRD7) was identified as a nuclear transcriptional regulatory factor. BRD7 functions as a tumor suppressor in multiple cancers, including nasopharyngeal carcinoma (NPC). In this study, we reported a novel mechanism of BRD7 in NPC progression. We demonstrated that the expression of miR-141 was remarkably increased in NPC tissues and was negatively correlated with the expression of BRD7 and the survival rate of NPC patients. Decreased expression levels of miR-141, including the primary, the precursor and the mature forms of miR-141, were found in BRD7-overexpressing HEK293, 5-8F and HNE1 cells compared the control cells, while there was no obvious effect on the expression levels of the two critical enzymes Drosha and Dicer. BRD7 can negatively regulate the promoter activity of miR-141, while no obvious binding site of BRD7 was found in the potential promoter region of miR-141. Moreover, ectopic expression of miR-141 can significantly promote cell proliferation and inhibit apoptosis in NPC, and rescuing the expression of miR-141 in BRD7-overexpressing NPC cells could partially reverse the tumor suppressive effect of BRD7 on cell proliferation and tumor growth *in vitro* and *in vivo*. Furthermore, the activation of the PTEN/AKT pathway mediated by the overexpression of BRD7 could be inhibited by rescuing the expression of miR-141, which accordingly results in the partial restoration of cell proliferation and tumor growth. Our findings demonstrate that the BRD7/miR-141/PTEN/AKT axis has critical roles in the progression of NPC and provide some promising targets for the diagnosis and treatment of NPC.

Nasopharyngeal carcinoma (NPC) represents a leading form of malignant head and neck cancer in Southeast Asia, especially in South-Eastern China. Although patient prognosis has improved markedly throughout past decades,^1^ most NPC patients are still diagnosed with advanced stage NPC.^2^ Therefore, it is essential for us to identify sensitive biomarkers and drug targets contributing to early diagnosis and improving treatment outcomes for NPC patients in the early stages. However, the molecular mechanisms regulating the initiation of NPC and promoting the malignant progression of NPC remain obscure.

Bromodomain containing (BRD7) is ubiquitously expressed in different tissue types^3^ and is predominantly localized in the nucleus.^4^ BRD7, serving as a transcriptional regulation factor, can modulate chromatin remodeling^5, 6^ and influence gene regulation by protein–protein interactions.^7, 8, 9, 10^ As a tumor suppressor, BRD7 is downregulated in multiple cancers, such as oral squamous cell carcinomas, epithelial ovarian carcinoma and NPC.^11, 12, 13^ Additionally, accumulating evidence indicates that BRD7 has critical roles in cell-cycle progression and survival in tumorigenesis^14, 15^ and regulates crucial signaling for cancer progression, such as Ras/Raf/MEK/ERK, Rb/E2F and PI3K/AKT signaling.^16, 17, 18^ However, the mechanism underlying the tumor suppressive effect of BRD7 in NPC progression has not yet been clearly explored.

MicroRNAs (miRNAs) represent a class of small functional non-coding RNAs (~20 nucleotides) that are involved in a wide range of biological processes such as cell proliferation, differentiation, apoptosis and motility by post-transcriptionally repressing gene expression.^19, 20, 21, 22^ miR-141 is a member of the miR-200 family. Deregulation of miR-200 family members in cancers has been associated with the growth, apoptotic response and regulation of metastasis.^23, 24, 25^ However, numerous studies have yielded conflicting results regarding the role of miR-141 in tumor progression of different cancer types.^26, 27, 28, 29^ In our previous work, we have demonstrated that miR-141 was upregulated in NPC tissues and downregulated by the knockdown of c-Myc in NPC cells.^30^ Interestingly, as a direct target gene of c-Myc,^31^ BRD7 can also downregulate the expression of miR-141 in NPC cells. Therefore, we hypothesized that miR-141 might be involved in BRD7-mediated cell proliferation and tumor formation in the progression of NPC.

In this study, we detected the level of miR-141 and BRD7 in NPC tissues and explored the correlation between their expression and the progression of NPC. Moreover, we performed rescue assays in BRD7-overexpressing NPC cells to investigate the role of miR-141 on cell proliferation and tumor growth *in vitro* and *in vivo*. Furthermore, we investigated the molecular mechanism underlying the function of the BRD7/miR-141 axis in cell proliferation and tumor growth of NPC. Our findings demonstrate that BRD7/miR-141/PTEN/AKT constitutes a signaling axis that has critical roles in NPC progression, and these findings provide some promising targets that could contribute to the diagnosis and treatment of NPC.

## Results

### miR-141 expression was negatively correlated with BRD7 and survival in human NPC patients

**Figure 1:**
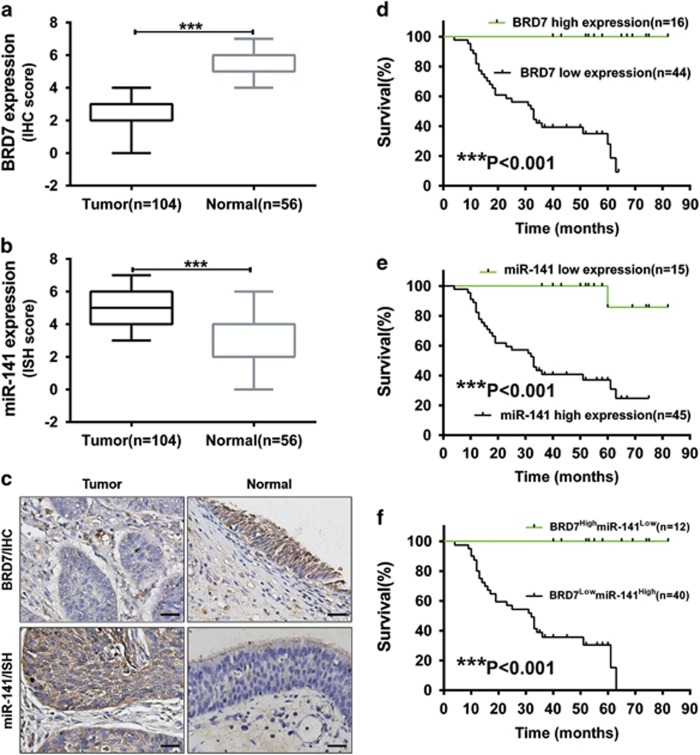
Increased expression of miR-141 was negatively correlated with BRD7 and patients' survival in human NPC. (**a**) BRD7 protein expression was analyzed by IHC (**c**, upper panel). (**b**) miR-141 expression was analyzed by ISH (**c,** lower panel). Box diagram of BRD7 and miR-141 expression in NPC (Tumor) (*N*=104) and non-cancerous nasopharyngeal control (Normal) (*N*=56) tissues, respectively. The whiskers represent the minimum and maximum values for each group. (**c**) Original magnification, × 200; the size bars represent 25 *μ*m. (**d**–**f**) Kaplan–Meier analysis to plot the overall survival (OS) curve of 60 NPC patients by expression of BRD7 and miR-141. Clinicopathological characteristics and statistical significance were assessed using the log-rank test. Kaplan–Meier curves showed worse survival rates in NPC patients with low BRD7 protein expression and high miR-141 expression compared with the related controls, respectively. ***P<0.001

To confirm the role of BRD7 and miR-141 in NPC, we detected their expression levels in NPC tissues derived from 104 patients and 56 healthy donors varying in age, sex and histological stage using immunohistochemical (IHC) staining and *in situ* hybridization (ISH) (Table 1, Figure 1c). As a result, most patients exhibited a significant decrease in the expression of the BRD7 protein and an increase in the level of miR-141 when compared with the normal control tissues (Figures 1a and b), and the expression level of miR-141 was negatively correlated with the protein level of BRD7 in NPC patients (*P*<0.001, Table 2). Moreover, we assessed the association between miR-141 level and prognosis in a cohort of 60 NPC tissues that contain survival information from the total of 104 NPC samples. The results showed that low BRD7 and high miR-141 expression in NPC patients revealed a poorer survival rate, respectively (Figures 1d and e). Simultaneously, high BRD7 and low miR-141 expression together revealed a favorable survival in NPC patients (Figure 1f). These findings indicate that both BRD7 and miR-141 might be involved in NPC progression and that BRD7 might be a negative regulator of miR-141 in NPC.

### miR-141 was downregulated in BRD7-overexpressing cell lines

**Figure 2:**
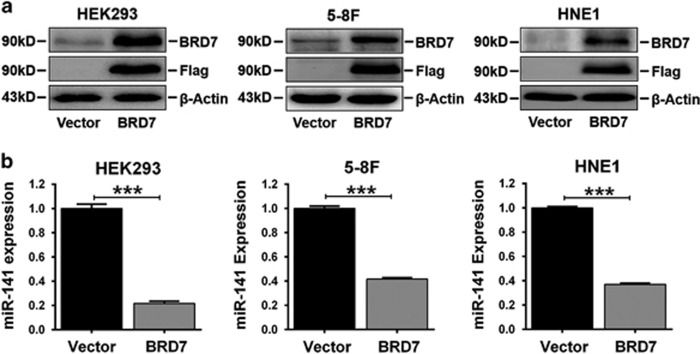
miR-141 was downregulated by BRD7 overexpression in HEK293, 5-8F and HNE1 stable cell lines. (**a**) Western blot using antibodies specific for BRD7 and Flag tag confirmed the exogenous BRD7 protein levels. *β*-Actin served as an internal control. (**b**) Mature miR-141 expression was detected by qRT-PCR. U6 served as an internal control. The error bars are represented as the mean±S.E.M. ***P<0.001. All experiments were performed in triplicate

To confirm the results observed in the NPC samples, we explored the relationship between the expression of miR-141 and BRD7 by stably transfecting a BRD7 expression construct into the HEK293, 5-8F and HNE1 cells. The expression of exogenous BRD7 was confirmed by western blot (Figure 2a). Then, we examined the effect of BRD7 overexpression on mature miR-141 expression. As a result, decreased mature miR-141 expression was detected in all of these BRD7-overexpressing cells when compared with the control cells (Figure 2b). These findings indicate that BRD7 functioned as a critical negative regulator in the process of miR-141 biogenesis.

### BRD7 transcriptionally downregulates miR-141 by repressing its promoter activity

**Figure 3:**
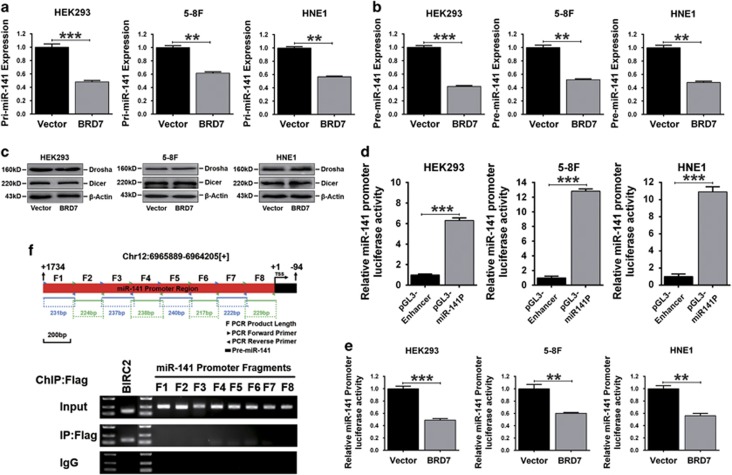
BRD7 downregulated the expression of miR-141 by repressing its promoter activity in HEK293, 5-8F and HNE1 stable cell lines. (**a**) The expression of pri-miR-141 and (**b**) pre-miR-141 was determined by qRT-PCR. GAPDH and U6 served as internal controls for pri-miR-141 and pre-miR-141, respectively. (**c**) Drosha and Dicer protein levels were detected by western blot. *β*-Actin served as an internal control. (**d**) The dual-luciferase reporter system assay evaluated the miR-141 potential promoter activity in HEK293, 5-8F and HNE1 cell lines and (**e**) in BRD7-overexpressing stable cell lines, normalized to pRL-TK. (**f**, upper panel) A schematic diagram showing the location of the miR-141 potential promoter region on chromosome 12 and the locations of the PCR primer pairs for the ChIP assay, and (**f**, lower panel) ChIP-PCR analysis for binding fragments of BRD7 within the miR-141 potential promoter region. BIRC2 was used as a positive control. F1–F8 represent the eight fragments in the miR-141 potential promoter of the PCR product. (**a**, **b**, **d** and **e**) The error bars are presented as the mean±S.E.M. **P<0.01, ***P<0.001. All experiments were performed in triplicate

miRNA biogenesis is a complex process including transcription and slicing of the primary transcript (pri-miRNA) or precursor miRNA (pre-miRNA).^32^ Therefore, to further explore the role of BRD7 in the process of miR-141 biogenesis, we first detected the levels of pri-miR-141 and pre-miR-141 in BRD7-overexpressing cell lines. The quantitative real-time PCR (qRT-PCR) data showed that pri-miR-141 and pre-miR-141 levels significantly decreased when BRD7 was overexpressed in HEK293, 5-8F and HNE1 cells (Figures 3a and b). Then, the expression levels of Drosha and Dicer, which are involved in the process of miRNA enzymatic processing, were detected. As a result, no significant changes were observed in the protein levels of Drosha and Dicer in BRD7-overexpressing cell lines (Figure 3c). These results suggest that BRD7 might not participate in miR-141 processing but may possibly be involved in the transcriptional regulation of miR-141.

To determine whether miR-141 was transcriptionally downregulated by BRD7, the ~1.7-kb length of the miR-141 potential promoter region was cloned into a pGL3-Enhancer vector (pGL3/miR-141P). The dual-luciferase reporter assays showed that the luciferase activity of the miR-141 promoter was significantly increased in HEK293, 5-8 F and HNE1 cells when compared with the empty pGL3-Enhancer vector-transfected cells (Figure 3d), revealing that the 1.7-kb region covers the miR-141 core promoter and possesses promoter activity. Beyond that, significant repression of the activity of the promoter was observed in HEK293/BRD7, 5-8F/BRD7 and HNE1/BRD7 cells compared with control cells (Figure 3e), demonstrating that BRD7 could significantly repress the miR-141 promoter activity.

Considering that BRD7 can act as a transcriptional regulator,^5, 10, 16, 33^ we investigated whether BRD7 could bind directly to the miR-141 potential promoter region. The 1.7-kb promoter region was divided into eight fragments using the DNA walking method, and each of the fragments was approximately 220–240 bp in length. The ChIP-PCR analysis showed that BRD7 could directly bind to the transcriptional regulation region of BIRC2, which was used as a positive control in this experiment,^34^ but could not bind to any of the eight fragments within the miR-141 promoter region (Figure 3f). These results suggest that BRD7, as a negative regulator, is involved in the transcriptional regulation of miR-141 in an indirect manner.

### miR-141 promotes cell proliferation and inhibits apoptosis in NPC cells

**Figure 4:**
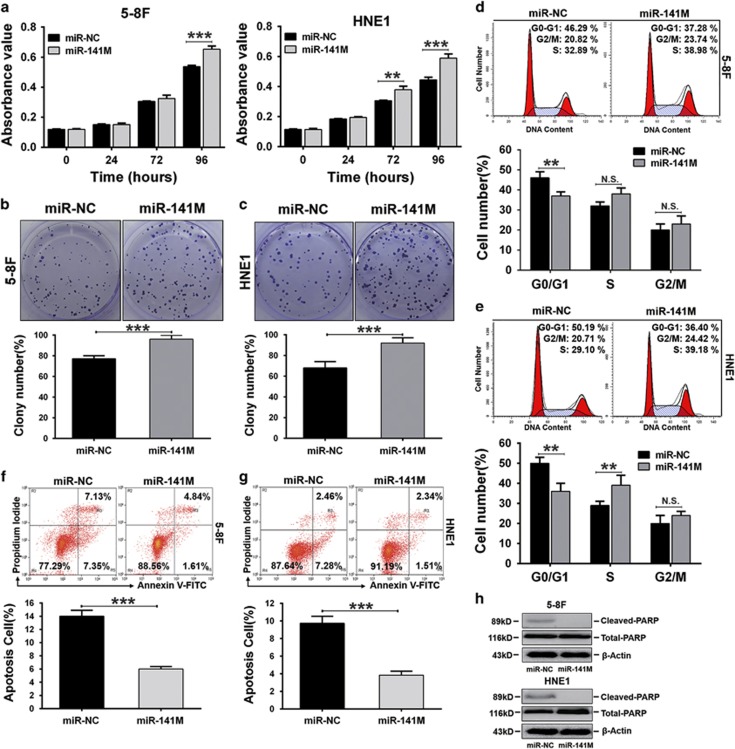
miR-141 promoted cell proliferation and inhibited apoptosis of 5-8F and HNE1 cell lines. (**a**) MTT assays of cells transfected with miR-141 mimic (miR-141M) or negative control (miR-NC). (**b** and **c**) Colony-forming assay images (upper panel) and quantification of colony number/well (lower panel). (**d** and **e**) Cell-cycle analysis by flow cytometry. (**f** and **g**) Annexin V-FITC and PI double staining analysis of cell apoptosis by flow cytometry. (**h**) Expression of the apoptosis marker cleaved-PARP (c-PARP) was determined by western blot. *β*-Actin served as an internal control. (**a**–**g**) The error bars are presented as the mean±S.E.M. **P<0.01, ***P<0.001, NS, no significance. All experiments were performed in triplicate

To confirm the role of miR-141 in NPC progression, we transfected miR-141 mimic into the 5-8F and HNE1 cell lines. The expression of exogenous miR-141 was confirmed by qRT-PCR (Supplementary Figure 1). We then examined the effect of miR-141 overexpression on cell proliferation and apoptosis. MTT assays showed that overexpression of miR-141 promotes cell proliferation in both 5-8F and HNE1 cell lines (Figure 4a), which was further confirmed by colony-forming assays. miR-141-overexpressing 5-8F and HNE1 (miR-141M) cells formed approximately 125 and 135% of the colonies of their negative controls (miR-NC), respectively (Figures 4b and c). Therefore, we further analyzed the effect of miR-141 overexpression on cell-cycle progression. The overexpression of miR-141 caused a significant decrease in the G0/G1 population, followed by an increase in the S-phase or G2/M cell fractions in 5-8 F and HNE1 cells (Figures 4d and e) when compared with control cells, respectively.

miR-141 was reported to be not only associated with the control of growth but also associated with the apoptotic response in some tumors.^35, 36^ Therefore, we also examined the effect of miR-141 overexpression on cell apoptosis by conducting Annexin V-FITC/propidium iodide (PI) double staining followed by flow-cytometry analysis, and we measured the expression of cleaved-PARP (c-PARP) protein by western blot when the cells were serum starved for 24 h. Annexin V-FITC/PI double staining analysis showed that both 5-8F and HNE1 cells transfected with miR-141 mimic had a significantly lower percentage of apoptotic cells compared with negative control cells (Figures 4f and g). Consistent with the flow-cytometry analysis results, the western blot assays revealed that the expression of c-PARP was not detected in the miR-141 mimic-transfected cells, but it was present in the control cells (Figure 4h). Taken together, these data confirmed that miR-141 promoted cell proliferation and inhibited apoptosis in both 5-8F and HNE1 cells and functioned as an oncomiRNA in NPC cells.

### Restoring the miR-141 level can reverse the effect of BRD7 on cell proliferation and apoptosis in NPC cells

**Figure 5:**
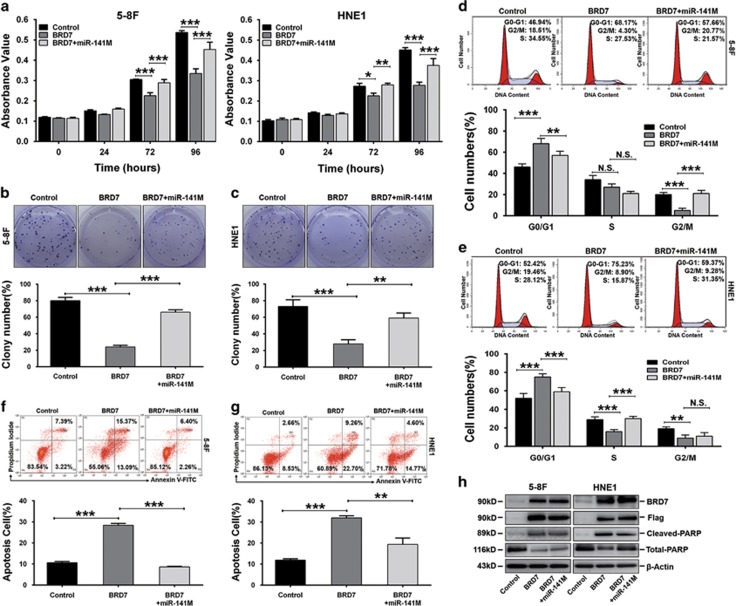
BRD7 inhibited cell proliferation and promoted apoptosis by downregulating miR-141 expression in 5-8F and HNE1 cells. (**a**) MTT assays of cells transfected with miR-141M or miR-NC. (**b** and **c**) Colony-forming assay images (upper panel) and quantification of colony number/well (lower panel). (**d** and **e**) Cell-cycle analysis by flow cytometry. (**f** and **g**) Annexin V-FITC and PI double staining analysis of cell apoptosis by flow cytometry. (**h**) Expression of the apoptosis marker c-PARP was determined by western blot. *β*-Actin served as an internal control. (**a**–**g**) The error bars are presented as the mean±S.E.M. Control: vector+miR-NC, BRD7: BRD7+miR-NC. **P<0.01, ***P<0.001, NS, no significance. All experiments were performed in triplicate

BRD7, as a tumor suppressor, could inhibit cell-cycle progression and initiate apoptosis; meanwhile, BRD7 could downregulate oncomiR-141 expression. Therefore, we investigated whether the effect of BRD7 on cell proliferation and apoptosis was mediated by its negative transcriptional regulation of miR-141. The rescue assays were performed by transfecting 5-8F/BRD7 and HNE1/BRD7 cells with miR-141 mimic. The restoration of miR-141 was confirmed by qRT-PCR assays (Supplementary Figure 2). To determine the impact of miR-141 restoration on cell proliferation, we performed MTT assays. As expected, restoring the miR-141 level in BRD7-overexpressing 5-8F and HNE1 cells (BRD7+miR-141) remarkably promoted cell proliferation compared with BRD7-overexpressing cells (BRD7) (Figure 5a), and the promotion of proliferation was confirmed using colony-forming assays (Figures 5b and c). Furthermore, we analyzed the effect of miR-141 restoration on cell-cycle progression, and the results showed that restoring the expression of miR-141 caused a significant decrease in the G0/G1 population that was associated with an increase in the S-phase or G2/M cell fractions in BRD7-overexpressing 5-8F and HNE1 cells (Figures 5d and e), respectively, when compared with BRD7-overexpressing control cells.

We also investigated the effect of the restoration of miR-141 expression on apoptosis in BRD7-overexpressing NPC cells. The NPC cells were stained with Annexin V-FITC and PI followed by flow-cytometry analysis after the cells were serum starved for 24 h. The results showed that the percentage of apoptotic cells significantly increased in the 5-8 F/BRD7 and HNE1/BRD7 cells when compared with the negative controls, while the percentage of apoptotic cells significantly recovered after the expression of miR-141 was restored in the 5-8 F/BRD7 and HNE1/BRD7 cells (Figures 5f and g). Consistent with the results from the flow-cytometry assays, the expression of c-PARP protein detected by western blot showed that treatment with serum starvation resulted in a significant decrease in the protein level of c-PARP when the expression of miR-141 was restored in BRD7-overexpressing cells compared with the BRD7-overexpressing cells (Figure 5h). All of these findings showed that restoring the expression of miR-141 could at least partially reverse the tumor suppressive effect of BRD7 on cell proliferation and apoptosis *in vitro* and demonstrate that BRD7, as a tumor suppressor, has a critical role in cell-cycle arrest and initiation of apoptosis through negative transcriptional regulation of miR-141 in NPC progression.

### miR-141 can rescue the tumor suppressive effect of BRD7 on tumor growth *in vivo*

**Figure 6:**
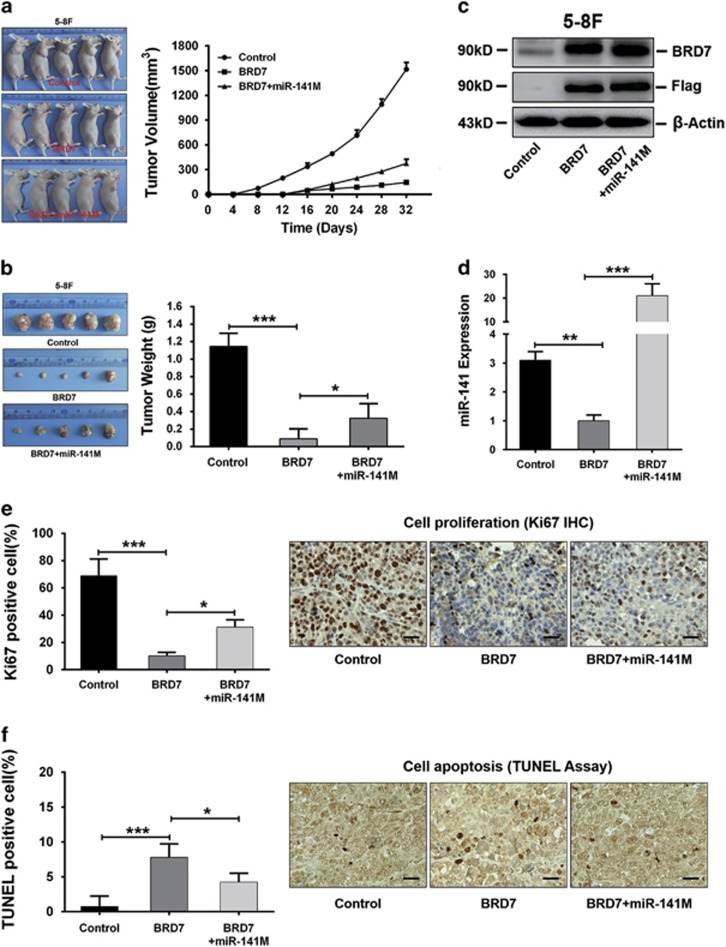
BRD7 inhibited tumor growth by downregulating miR-141 expression *in vivo*. (**a**) Images (left panel) and tumor growth curve (right panel) of the 5-8 F xenograft model in nude mice. (**b**) Tumor images (left panel) and weight quantification (right panel) (*N*=5). (**c**) Western blot and (**d**) qRT-PCR assay confirmed the expression of the BRD7 protein and of mature miR-141 in xenograft tumor tissues, respectively. *β*-Actin and U6 served as internal controls, respectively. (**e**) IHC (DAB staining) for quantification of the proliferation marker Ki-67 (left panel) and images (right panel) and (**f**) TUNEL (DAB staining) for quantification of apoptosis (left panel) and images (right panel). Three tumors were analyzed per group using immunohistochemistry for the molecules mentioned above. (**e** and **f**) Original magnification, × 200; the size bars represent 25 *μ*m. (**b**, **d**, **e** and **f**) The error bars are presented as the mean±S.E.M. Control: Vector+miR-NC, BRD7: BRD7+miR-NC. *P<0.05, **P<0.01, ***P<0.001

To corroborate our *in vitro* findings, we performed *in vivo* experiments with xenograft tumor models in nude mice. 5-8 F/BRD7 and 5-8 F/Vector cells transfected with miR-141 mimic or negative control were injected subcutaneously into the flank of 6-week-old female nude mice. All xenograft model mice were killed on day 32 to examine the final tumor volume. The growth rate (Figure 6a) and weight (Figure 6b) of the xenograft tumors with miR-141 restoration were significantly increased compared with those of the 5-8 F/BRD7 group. In addition, the expression of BRD7 and miR-141 in xenograft tumor tissues was confirmed by western blot (Figure 6c) and by qRT-PCR assays (Figure 6d), respectively.

To further confirm the effect of the restoration of miR-141 on tumor growth and apoptosis *in vivo*, we analyzed the percentage of cells expressing the proliferation marker Ki-67 and the percentage of TUNEL-positive cells, respectively. Consistent with the *in vitro* results, the restoration of miR-141 expression caused a significant increase in the number of Ki-67+ cells and a marked decrease in the number of TUNEL+ cells in BRD7-overexpressing xenograft tumors when compared with BRD7-overexpressing controls (Figures 6e and f), respectively. These *in vivo* findings correspond with those of our *in vitro* studies. All of these *in vitro* and *in vivo* results demonstrate that the tumor suppressive effect of BRD7 on tumor growth in NPC progression occurred at least partially through its negative transcriptional regulation of miR-141.

### Downregulation of miR-141 by BRD7 inhibits the PTEN/AKT pathway in NPC

**Figure 7:**
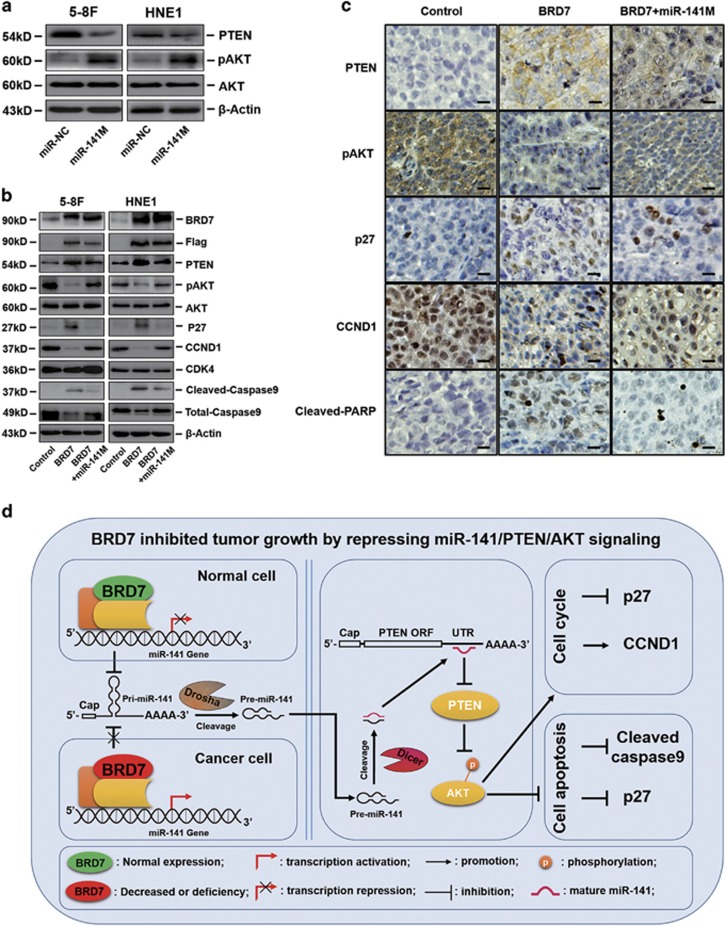
BRD7 inhibited tumor growth by repressing miR-141/PTEN/AKT signaling. (**a**) PTEN and phosphorylated AKT protein expression levels in miR-141 mimic-transfected 5-8F and HNE1 cells were analyzed by western blotting, and (**b**) significantly differently expressed proteins downstream of PTEN/AKT signaling were involved in cell-cycle progression (p27, CCND1 and CDK4) and survival (cleaved-caspase-9, total-caspase-9 and p27) when miR-141 expression was restored in BRD7-overexpressing 5-8F and HNE1 stable cell lines, respectively. *β*-Actin served as an internal control. (**c**) IHC (DAB staining) for PTEN, p-AKT, p27, CCND1 and cleaved-PARP in the 5-8F xenograft model. Three tumors were analyzed per group using immunohistochemistry for the molecules mentioned above. Original magnification, × 200; the size bars represent 25 *μ*m. Control: Vector+miR-NC, BRD7: BRD7+miR-NC. (**d**) A schematic map showing the tumor suppressive mechanism of BRD7 that occurs by repressing the miR-141/PTEN/AKT pathway in NPC

PTEN negatively regulates the intracellular level of PIP3, functions as a tumor suppressor by negatively regulating the AKT signaling pathway, and is involved in the regulation of growth and apoptosis in various cancers.^37, 38^ Additionally, we have previously demonstrated that miR-141 could directly target PTEN and downregulate PTEN protein levels in NPC cells.^30, 39^ In this study, the overexpression of BRD7 in either 5-8F or HNE1 cells led to an increase in the protein level of PTEN and a decrease in the protein level of p-AKT (Figure 7a). Furthermore, the restoration of miR-141 reduced the protein level of PTEN and increased the expression of p-AKT in BRD7-overexpressing NPC cells compared with BRD7-overexpressing controls (Figure 7b). These results indicated that BRD7 upregulated the expression of the PTEN protein and inhibited the phosphorylation level of p-AKT by transcriptionally downregulating miR-141 expression *in vitro*.

Notably, IHC staining of tumor sections of xenografts demonstrated that the overexpression of BRD7 significantly induced the expression of PTEN and decreased the phosphorylation of p-AKT in the 5-8F/BRD7 tumors compared with the 5-8F/mock tumors (Figure 7c). Collectively, these data demonstrate that BRD7 can inhibit the phosphorylation level of p-AKT by upregulating the PTEN protein level via transcriptionally downregulating the expression of miR-141 *in vitro* and *in vivo*.

To identify effectors responsible for the phenotypes observed *in vitro* and *in vivo*, we examined alterations in the expression of key components downstream of the BRD7/miR-141/PTEN/AKT axis. Western blotting revealed that the restoration of miR-141 expression in BRD7-overexpressing cells resulted in a decrease in p27 and an increase in CCND1, while CDK4 levels remained unaltered (Figure 7b). We also detected the effectors related to apoptosis downstream of the PTEN/AKT pathway. The data revealed that the expression of cleaved caspase-9 was decreased in BRD7-overexpressing cells upon restoration of miR-141 expression when compared with BRD7-overexpressed cells (Figure 7b). Furthermore, IHC staining of tumor sections of xenografts demonstrated that p27 and c-PARP were decreased, while CCND1 was increased in 5-8F/BRD7 tumors when miR-141 expression was restored compared with the miR-NC-transfected 5-8F/BRD7 tumors (Figure 7c). Collectively, these data showed that BRD7 inhibited cell proliferation and initiated apoptosis by downregulating the miR-141/PTEN/AKT pathway *in vitro* and *in vivo* (Figure 7d).

## Discussion

Accumulating evidence indicates that BRD7, as a tumor suppressor, has crucial roles in the progression of multiple cancers.^40, 41^ Here, we characterized the expression of BRD7 in NPC specimens and found that most NPC patients exhibit significant downregulation of the BRD7 protein, and low BRD7 protein levels were associated with poor prognosis of NPC patients. These findings further confirm that BRD7 functions as a tumor suppressor and has critical roles in NPC progression.

miRNAs, as a major category of small functional non-coding RNAs, can repress the expression of target genes at the post-transcriptional level^42, 43^ and can have vital roles in the progression of multiple cancers.^44, 45^ In this study, we report that miR-141 expression significantly increased in NPC tissues compared with normal controls, and high miR-141 levels inversely correlate with overall survival (OS) of NPC patients. We have previously shown that miR-141, as a potential oncomiRNA, was upregulated in NPC tissues and regulated some critical pathways, such as Rb/E2F, JNK2 and AKT signaling in NPC progression.^30^ Furthermore, miR-141 levels can be repressed by knockdown of c-Myc and SPLUNC1 overexpression *in vitro* and *in vivo*.^30, 39^ C-Myc, as a well-known transcription factor, can regulate the transcription of numerous target genes,^46, 47^ and BRD7 is one of the target genes directly downregulated by c-Myc.^31^ These results are also consistent with our findings above. Moreover, the expression of miR-141 and BRD7 is significantly inversely correlated in NPC patients, and low protein levels of BRD7 and high expression of miR-141 were associated with poor prognosis of NPC patients. All of these findings further demonstrate that miR-141, serving as an oncomiRNA, has critical roles in NPC progression.

BRD7 was mainly localized in the nucleus with a functional nuclear location signal (NLS),^4, 48^ and BRD7 functioned as a nuclear transcriptional regulation factor.^33, 49, 50^ In this study, we found that overexpression of BRD7 could downregulate pri-, pre- and mature miR-141 expression, while it did not affect the expression levels of Drosha and Dicer proteins, which are involved in miRNA enzymatic processing. Meanwhile, the overexpression of BRD7 significantly repressed the activity of the miR-141 potential promoter. These findings demonstrate that BRD7 transcriptionally downregulates miR-141. However, the ChIP-PCR assays indicated that there was not a BRD7-binding region in the miR-141 potential promoter. As a transcriptional regulation factor, BRD7 generally regulates the transcriptional activity of target genes by promoting protein–protein interactions and forming transcriptional complexes.^33, 51^ Therefore, considering the functioning model of BRD7 generally, we maintain that BRD7 might interact with some regulators involved in basal transcriptional regulation of miR-141 to repress its promoter activity and downregulate its expression.

miR-141 belongs to the miR-200 family, the members of which have critical roles in cancer development and progression.^52, 53^ In recent years, numerous studies have yielded conflicting results regarding the effect of miR-141 in different cancer types.^28, 54^ Here, we demonstrated that, in NPC progression, miR-141, functioning as an oncomiRNA, promotes cell proliferation and inhibits apoptosis. Furthermore, we found that restoring the level of miR-141 in BRD7-overexpressing NPC cells can partially reverse the effect of BRD7 on cell proliferation and apoptosis *in vitro* and *in vivo*, indicating that the effect of BRD7 in NPC progression is partially dependent on its negative transcriptional regulation of miR-141.

Our previous study reported that PTEN is a direct target of miR-141 and that the expression of the PTEN protein is downregulated by miR-141 in NPC cells.^30, 39^ Moreover, increasing evidence has indicated that aberrant activation of the PTEN/AKT pathway is essential for the development and progression of NPC.^55, 56^ Here, we confirmed that miR-141 decreases the protein level of PTEN in NPC cells and promotes the phosphorylation of AKT. In contrast, the overexpression of BRD7 in NPC cells resulted in an increase in PTEN protein and a decrease in phosphorylation of AKT. In addition, in BRD7-overexpressing NPC cells in which the expression of miR-141 was restored, we found that the effect of BRD7 on the PTEN/AKT axis was at least partially reversed *in vitro* and *in vivo*. Considering these findings, we further explored the differential expression of components downstream of the PTEN/AKT pathway that are responsible for cell-cycle progression and survival. Numerous studies have demonstrated that p27, CCND1 and caspase-9 are critical regulators downstream of the PTEN/AKT pathway that are responsible for cell-cycle progression and survival, respectively.^57,58,59^ In this study, we found that p27 and C-caspase-9 were notably upregulated and CCND1 was significantly downregulated in BRD7-overexpressing NPC cells, while the expression changes were the opposite when the miR-141 level was restored in BRD7-overexpressing NPC cells *in vitro* and *in vivo*. These findings further demonstrate that BRD7, as a critical negative regulator, could repress the miR-141/PTEN/AKT pathway in NPC progression.

In summary, we demonstrated that BRD7 represses PTEN/AKT signaling by transcriptionally downregulating miR-141 expression contributing to cell-cycle arrest and initiation of apoptosis in NPC. We also showed that the BRD7/miR-141 axis is significantly associated with the OS of NPC patients. All of these findings suggest that the expression of BRD7 and miR-141 might serve as sensitive biomarkers in the early diagnosis of NPC; meanwhile, inhibitors specific for miR-141 could provide a promising strategy for NPC therapy.

## Materials and Methods

### Ethical statement

In our study, ethical approval was obtained from the Ethics Review Committees/Institutional Review Boards of Central South University. NPC samples and non-cancerous nasopharyngeal tissues from healthy donors were collected at the Second Xiangya Hospital of Central South University (Changsha, China). Written informed consent was obtained from all patients and donors. All animal procedures were conducted in accordance with protocols approved by the Institutional Animal Care and Use Committee (IACUC) of Central South University.

### Tissue samples

NPC samples (*n*=104) and non-cancerous nasopharyngeal tissues (*n*=56) from healthy donors were collected at the Second Xiangya Hospital of Central South University (Changsha, China). The non-cancerous nasopharyngeal tissues were collected from independent patients with chronic inflammation of nasopharyngeal mucosa or polyps. The profile of clinicopathological characteristics of the NPC patients is shown in Table 1. The age of the NPC patients ranged from 19 to 77 years old, and 60 of the 104 patients had valid follow-up data. The longest survival time was 84 months. OS was defined as the time from diagnosis to the date of death or the date that the patient was last known to be alive. All tissue samples were immediately snap-frozen in liquid nitrogen and stored in a refrigerator at −80 °C. Tissues for immunohistochemistry were fixed in 4% paraformaldehyde and paraffin embedded. Clinicopathological data were reviewed, and TNM staging classification was based on the criteria of the American Joint Committee on Cancer (AJCC, 6th edition).

### Cell lines

The human NPC cell lines 5-8F and HNE1 were preserved in our laboratory, which were cultured in RPMI-1640 (Hyclone, Logan, UT, USA) supplemented with 10% fetal bovine serum (FBS) (Gibco-BRL, Invitrogen, Paisley, UK) in a humidified incubator with 5% CO_2_ at 37 °C. The embryonic kidney cell line HEK293 was also preserved in our laboratory and maintained in Dulbecco's modified Eagle's medium (DMEM) (Hyclone) with 10% FBS (Gibco) at 37 °C in a humidified atmosphere with 5% CO_2_.

### Construction of BRD7 expression vector and cell transfection

The expression vector encoding full-length open reading frame of human BRD7 with 3Flag tags (pIRESneo3/3Flag-BRD7) was constructed by Xu *et al.*^34^ The plasmids were transfected using Lipofectamine3000 Reagent (Invitrogen, Carlsbad, CA, USA). The stably transfected cells were screened under G418 (Invitrogen). Stable pool clones were obtained.

The hsa-miR-141 mimic (miR-141M) and negative control (miR-NC) were purchased from RiBoBio (Guangdong, China). Cells in logarithmic growth phase were trypsinized, counted and seeded in a 6-well plate to ensure 60–80% cell confluence on the next day for transfection. Transfection of cells with oligonucleotides was performed using Lipofectamine3000 Reagent (Invitrogen) according to the manufacturer's protocol at a final concentration of 50 nM.

### RNA isolation and qRT-PCR

Total RNA was isolated from tissue samples and cell lines using TRIzol reagent (Invitrogen) according to the manufacturer's protocol. The expression level of mature miR-141 and pre-miR-141 was evaluated using the miDETECT A Track miRNA qRT-PCR Kit (RiBoBio) following the manufacturer's protocol. The U6 small nuclear RNA (RNU6B) (RiBoBio) was used for normalization. The expression level of pri-miR-141 was measured by qRT-PCR according to the instructions of the SYBR Premix Ex Taq (TaKaRa, Dalian, China). The GAPDH mRNA level was used for normalization. The relative expression ratio of mature miR-141, pre-miR-141 and pri-miR-141 was calculated using the 2^−ΔΔCT^ method. The primer for mature miR-141 was 5′-TAACACTGTCTGGTAAAGATGG-3′ (forward). Primer for pre-miR-141 was 5′-TTGGATGGTCTAATTGTGAAGCTCC-3′ (forward). Primer pairs for U6 were 5′-ATTGGAACGATACAGAGAAGATT-3′ (forward) and 5′-GGAACGCTTCACGAATTTG-3′ (reverse). Primer pairs for pri-miR-141 were 5′-AGACCTCACCTGGCCTGTGGCC-3′ (forward) and 5′-GAACCCACCCGGGAGCCATCTT-3′ (reverse). Primer pairs for GAPDH were 5′-CGAGATCCCTCCAAAATCAA-3′ (forward) and 5′-TTCACACCCATGACGAACAT-3′ (reverse). PCRs of each sample were conducted in triplicate.

### Western blot analysis

Cells and tissue samples were lysed in RIPA buffer in the presence of Protease Inhibitor Cocktail and PhoSTOP (Roche, Basel, Switzerland). Protein was quantified using a BCA Protein Assay Kit (Pierce Biotechnology, Rockford, IL, USA). Protein (30–80 *μ*g) was separated by 10% sodium dodecyl sulfate polyacrylamide gel electrophoresis, and transferred onto polyvinylidene fluoride membranes (PVDF) (Millipore, Billerica, MA, USA). The membranes were blocked with 5% non-fat milk in Tris-buffered saline and then incubated with primary antibodies at 4 °C overnight. The primary antibodies used were anti-BRD7 (dilution 1:500; ProteinTech, Wuhan, China), anti-Flag (dilution 1:2000; Sigma, St. Louis, MO, USA), anti-*β*-actin (dilution 1:1000; Santa Cruz, CA, USA), anti-PARP (dilution 1:1000; CST, Danvers, MA, USA), anti-c-PRAP (dilution 1:1000; CST), anti-CCND1 (dilution 1:500; Santa Cruz), anti-CDK4 (dilution 1:500; Santa Cruz), anti-PTEN (dilution 1:500; Bioworld Technology, Atlanta, GA, USA), anti-pS473AKT (dilution 1:500; Bioworld Technology), anti-AKT (dilution 1:500; Bioworld Technology), anti-p27 (dilution 1:1000; CST), anti-caspase-9 (dilution 1:500; Santa Cruz) and anti-C-asepase-9 (dilution 1:500; Santa Cruz). Membranes were then washed three times in TBST solution for 10 min each time, and then incubated with secondary antibodies. Signals were detected by an enhanced chemiluminescence detection system as the manufacturer's protocol (Bio-Rad, Hercules, CA, USA).

### Construction of the reporter recombinant linked with miR-141 promoter and luciferase reporter assays

An ~1.7-kb fragment of the miR-141 promoter fused with Sac I and Hind III linkers was amplified by PCR using the following primers: 5′-TTTGAGCTCAAGAAGGAAGCAAACAAAGCCTGG-3′ (forward) and 5′-TTTAAGCTTACAGAGAACTACGGTGCGCG-3′ (reverse). After digestion with Sac I and Hind III, the fragment was cloned into the Sac I and Hind III sites of the pGL3-Enhancer luciferase vector (Promega, Madison, WI, USA) and named pGL3/miR-141P. Proper insertion was confirmed by sequencing. The cells were seeded in 24-well plates. After 24 h, the cells were transfected with pGL3/miR-141 or pGL3-Enhancer vector, together with the pRL-TK vector (Promega) containing Renilla luciferase. Transfection was performed using Lipofectamine 3000 (Invitrogen). Cells were harvested 36 h post transfection. Firefly and Renilla luciferase activities were measured using a Dual-luciferase reporter kit (Promega) according to the manufacturer's protocol. Firefly luciferase activity was normalized to Renilla luciferase activity.

### Chromatin immunoprecipitation assays

HEK293 was cross-linked in 1% formaldehyde for 10 min at 37 °C. DNA from fixed chromatin cells was then subjected to immunoprecipitation using a ChIP assay kit (Millipore) and antibodies against Flag or anti-mouse IgG according to the manufacturer's protocol. The purified DNA was used for the following PCR. On the basis of the requirement of the ChIP assay, the 1.7-kb promoter region of miR-141 was divided into eight fragments using the DNA walking method. Each of the fragments was approximately 220–240 bp length. The primer pairs for the detection of the eight fragments in the miR-141 promoter region were generated by Invitrogen as follows: F1: 5′-AAGCCTGGGAGAGAGAACAGCG-3′ (forward) and 5′-TCACAGCACAGCCCTCAGTG-3′ (reverse), F2: 5′-TGTGCTGTGAGGTGGGTCCG-3′ (forward) and 5′-TGTGCTGGGATCCCCTGAGG-3′ (reverse), F3: 5′-TCAGGGGATCCCAGCACAGG-3′ (forward) and 5′-TCTCCAACCTGCCTAGTCTCACC-3′ (reverse), F4: 5′-TGAGACTAGGCAGGTTGGAG-3′ (forward) and 5′-TGGCCTCCGCTCTTCCTCC-3′ (reverse), F5: 5′-AAGGAAGGAGGAAGAGCGGAG-3′ (forward) and 5′-TCTGAGCCACCTTCCCCTACC-3′ (reverse), F6: 5′-AAGGTGGCTCAGAGGCGGC-3′ (forward) and 5′-TTGGGTCAGGCAGCTTCAGG-3′ (reverse), F7: 5′-AAGCTGCCTGACCCAAGGTG-3′ (forward) and 5′-AACGCTCTCAGCTCAAGACG-3′ (reverse) and F8: 5′-TTGAGCTGAGAGCGTTGCAC-3′ (forward) and 5′-ACAGAGAACTACGGTGCGCG-3′ (reverse).

### Cell proliferation (MTT) and colony-forming assays

For MTT, 1000 5-8F and HNE1 cells were plated onto a 96-well plate. Plates were harvested daily. A total of 20 *μ*l of 5 mg/ml MTT (3-(4,5-Dimethylthiazol-2-yl)-2,5-diphenyltetrazolium bromide, a tetrazole) (Invitrogen) was added to each well, then cells were incubated for 4 h in 37 °C and carefully removed without disturbing the cells. A total of 150 *μ*l MTT solvent (4 mM HCl, 0.1% Nondet P-40 (NP40)) in isopropanol was added to each well, and the plate was shaken at room temperature for 10 min. The number of viable cells was determined by the measurement of the optical density difference between 450 and 600 nm using a Beckman microplate reader (Beckman, Brea, CA, USA).

For the colony-forming assay, approximately 150 cells per well were added to a 6-well plate, with three wells per sample. After a 14-day incubation, the cells were washed twice with PBS and stained with Giemsa solution (Beyotime, Beijing, China). Colonies containing more than 50 cells were counted as 1 positive colony. The plate clone formation efficiency was calculated as (number of colonies/number of cells inoculated)  × 100%. All experiments were performed in triplicate.

### IHC staining and ISH

For IHC staining, paraffin sections, 4 *μ*m in thickness, were baked for 1 h at 65 °C. After deparaffinization and rehydration, antigen retrieval was performed by boiling in 0.01 M citrate buffer (pH 6.0) in a domestic microwave oven at full power (750 Watts) for 20 min. After inhibition of endogenous peroxidase activity for 30 min with methanol containing 0.3% H_2_O_2_, the sections were blocked with 2% bovine serum albumin in PBS for 30 min and then incubated with anti-BRD7 (dilution 1:100; Proteintech), anti-PTEN (dilution 1:100; Bioworld Technology), anti-pS473AKT (dilution 1:100; Bioworld Technology), anti-p27 (dilution 1:100; CST), anti-CCND1 (dilution 1:100; Santa Cruz Biotechnology) and anti-c-PRAP (dilution 1:100; CST) antibodies. The immune complex was visualized by the MaxVison HRP-polymer IHC Kit Detection System, Peroxidase/DAB, Rabbit/Mouse (MaxVison, Fuzhou, China) according to the manufacturer's protocol. The nuclei were counterstained with hematoxylin (Beyotime). Positive control slides were included in every experiment in addition to the internal positive control. The specificity of the antibody was determined using a matched IgG isotype antibody as a negative control.

For ISH staining, the mature Hsa-miR-141-specific probe and negative control (Scramble) were purchased from Sangon (Sangon Biotech, Shanghai, China). An *in situ* hybridization kit (Boster, Wuhan, China) was used for hybridization of the probe according to the manufacturer's instructions.

### Scores for IHC and ISH analysis

IHC and ISH staining were evaluated, at × 200 magnification using light microscopy, independently by two pathologists who were blinded to the clinicopathological data. A semiquantitative evaluation of BRD7 protein and mature miR-141 was performed using a method described in the literature^60^ that was based on the staining intensity and extent of staining as follows: staining intensity for BRD7 and miR-141 was scored as 0 (negative), 1 (weak), 2 (moderate) and 3 (strong). Staining extent was scored as 0 (0), 1 (1–25), 2 (26–50), 3 (51–75) and 4 (76–100%), depending on the percentage of positively stained cells. The sum of the staining intensity and the staining extent scores ranged from 0 to 7, and the cutoff levels for BRD7 and miR-141 were chosen on the basis of a measure of heterogeneity using the log-rank test with respect to OS. An optimal cutoff level was identified as follows: a staining index score of 0–2 was used to define tumors with low expression and staining index scores of 3–7 indicated high expression of BRD7 protein and mature miR-141. The agreement between the two evaluators was 95%, and all scoring discrepancies were resolved by discussion between the two evaluators.

### 5-8F tumor xenograft model

A total volume of 100 *μ*l of cells (5 × 10^6^ cells) transfected with miR-141 mimic or negative control were inoculated subcutaneously into the right flanks of 6-week-old female nude mice. Mice were checked every 4 days, and tumor nodules were measured with a caliper. Tumor volume was evaluated using the following formula: volume=(width+length)/2 × width × length × 0.5236. Tumor growth curves were calculated. The three experimental groups were killed after 32 days. All tumor grafts were excised, weighed, harvested, fixed and embedded. The anti-Ki-67 antibody (dilution 1:100, Bioworld) was used to detect the proliferation marker Ki-67 using immunohistochemistry procedures, and TUNEL assay was used to detect apoptosis *in situ* with paraffin-embedded xenograft sections using the DeadEnd Colorimetric TUNEL Detection Kit (Promega) according to the manufacturer's protocols. Samples were observed using an Olympus microscope (Olympus, Tokyo, Japan). The proliferative and apoptosis index scores were measured as the mean percentage of nuclei that stained positive for Ki-67 and TUNEL cells in 10 different × 200 fields.

### Statistical analysis

The relationships between the expression levels of miR-141, the BRD7 protein and clinicopathological characteristics in NPC were tested using the chi-square test. The Spearman's rank correlation coefficient was used to assess the significance of the association among expression of BRD7 and miR-141 in NPC. Kaplan–Meier analysis was performed for OS curves, and statistical significance was assessed using the log-rank test. The differences between the groups were analyzed using the Student's *t-*test when there were only two groups or using one-way ANOVA when there were more than two groups. All statistical analyses were performed using the SPSS software (SPSS, Chicago, IL, USA). A two-tailed value of *P*<0.05 was considered statistically significant.

## Figures and Tables

**Table 1:** Association between expression of BRD7, mature miR-141 and NPC clinical pathological features (*N*=104)

	**BRD7**			**miR-141**			**BRD7/miR-141**^a^		
**Characteristics (*N*)**	**H (%)**	**L (%)**	***P*-value**	**H (%)**	**L (%)**	***P*-value**	**H-L (%)**	**L-H (%)**	***P*-value**
*Age (year)*									
≤40 (*n*=27)	10 (37.0)	17 (63.0)	0.576	18 (66.7)	9 (33.3)	0.452	7 (25.9)	16 (59.3)	0.764
>40 (*n*=77)	24 (31.2)	53 (68.8)		45 (58.4)	32 (41.6)		19 (24.7)	37 (48.1)	

*Gender*									
Female (*n*=23)	6 (26.1)	17 (73.9)	0.384	13 (56.5)	10 (43.5)	0.652	5 (21.7)	12 (52.2)	0.759
Male (*n*=81)	29 (35.8)	52 (64.2)		50 (61.7)	31 (38.3)		21 (25.9)	42 (51.9)	

*Histological type*									
DNC (*n*=6)	2 (33.3)	4 (66.7)	0.986	3 (50.0)	3 (50.0)	0.585	2 (33.3)	3 (50.0)	0.776
UDNC (*n*=98)	33 (33.7)	65 (66.3)		60 (61.2)	38 (38.8)		26 (26.5)	51 (52.1)	

*Clinical stages*									
Stage I (*n*=14)	8 (57.1)	6 (42.9)	0.004	5 (35.7)	9 (64.3)	0.003	6 (42.9)	3 (21.4)	0.0004
Stage II (*n*=33)	14 (42.4)	19 (57.6)		13 (39.4)	20 (60.6)		12 (36.4)	12 (36.4)	
Stage III (*n*=39)	12 (30.8)	27 (69.2)		27 (69.2)	12 (30.8)		8 (20.5)	24 (61.5)	
Stage IV (*n*=18)	2 (11.1)	16 (88.9)		13 (72.2)	5 (27.8)		1 (55.6)	13 (72.2)	

Abbreviations: DNKC, differentiated non-keratinized nasopharyngeal carcinoma; UDNC, undifferentiated non-keratinized nasopharyngeal carcinoma; H, high expression; L, low expression

a H-L, High expression of BRD7 and low expression of miR-141; L-H, Low expression of BRD7 protein and high expression of miR-141

**Table 2:** BRD7 and miR-141 exhibit inverse expression pattern in human NPC patients (*N*=104)

	**BRD7 expression (IHC)**		
	**High**	**Low**	***P*-value**
*miR-141 expression (ISH)*			
Low	27	19	<0.001
High	6	52	

## Abbreviations

BRD7: Bromodomain containing 7
miR-141: microRNA-141
NPC: nasopharyngeal carcinoma
IHC: immunohistochemical
ISH: *in situ* hybridization
pri-miR-141: primary miR-141
pre-miR-141: precursor miR-141
PTEN: phosphatase and tensin homolog deleted on chromosome ten
