# Supplementary material for: miR-141 is involved in BRD7-mediated cell proliferation and tumor formation through suppression of the PTEN/AKT pathway in nasopharyngeal carcinoma
Source: Cell Death Dis. 2016 Mar 24;7(3):e2156–. doi: 10.1038/cddis.2016.64 (PMC4823963; doi:10.1038/cddis.2016.64)
Supplement: Supplementary Figures [file cddis201664x1.doc]

**Supplementary Figures**

**Supplementary Figure 1**

**
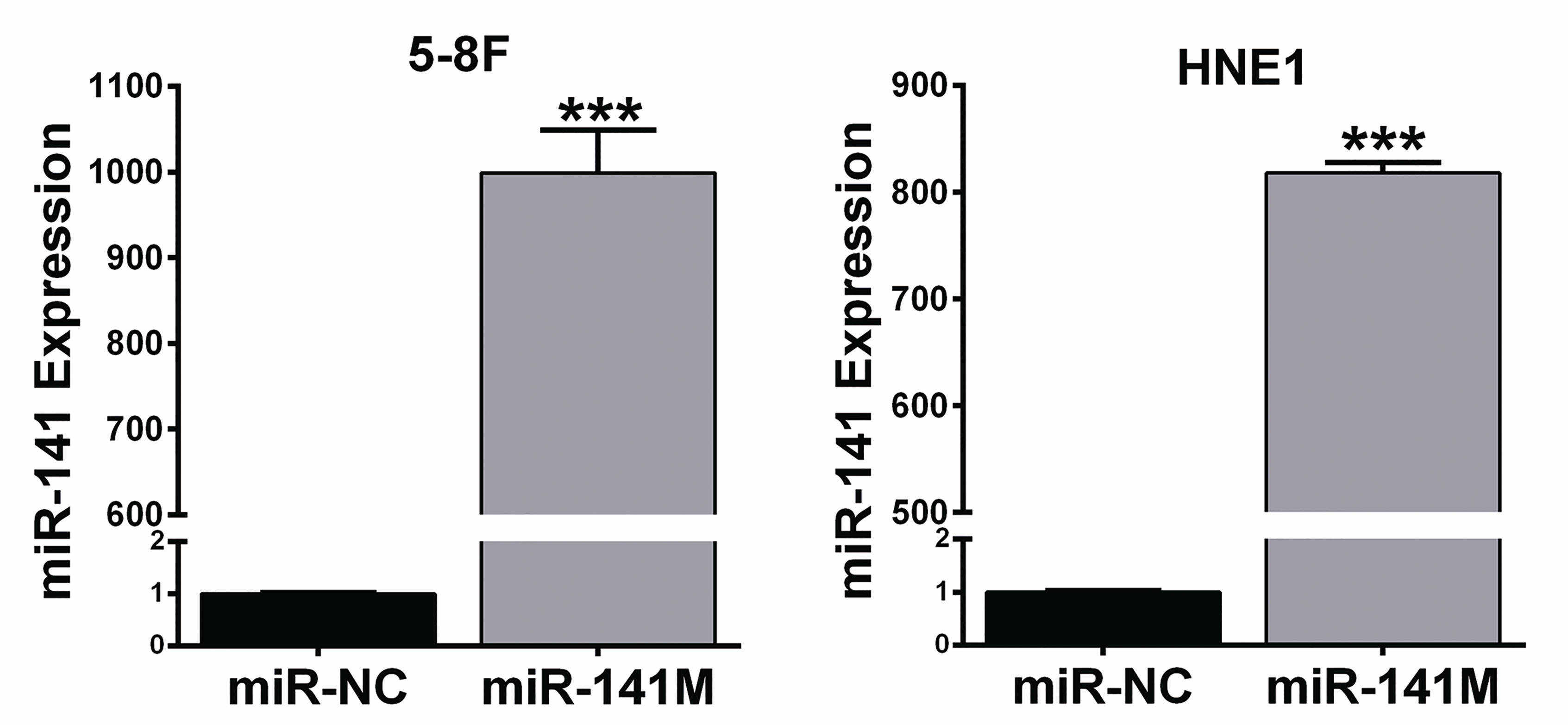
**

**Supplementary Figure 1. The expression of mature miR-141 in miR-141-overexpressing cell models of 5-8F and HNE1.** qRT-PCR detection confirmed the expression of mature miR-141 in the miR-141 mimic (miR-141M) or negative control (miR-NC) transfected NPC cells. U6 serve as an internal control. The error bars are presented as the mean ± S.E.M. ***P<0.001. All experiments were performed in triplicate.

**Supplementary Figure 2**

**
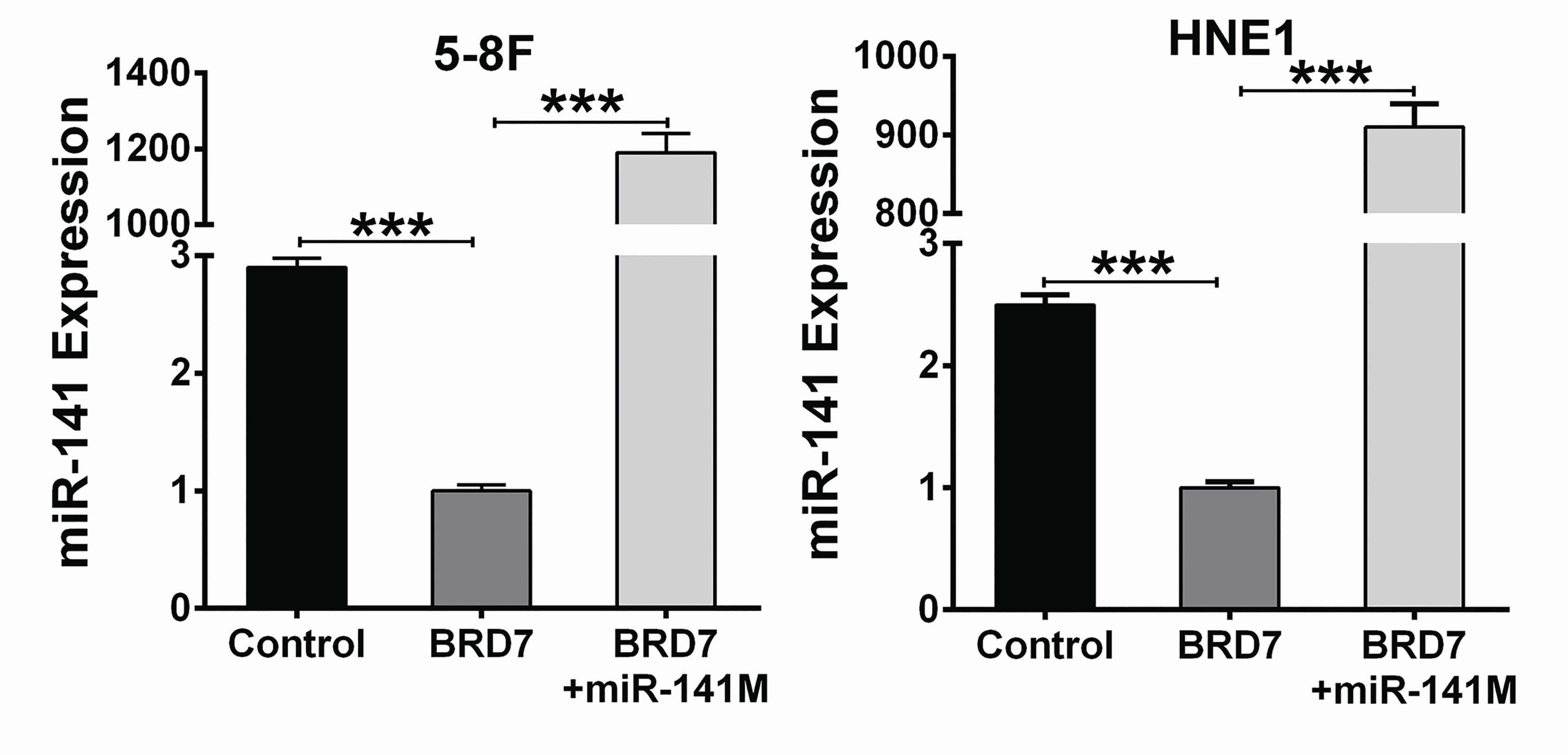
**

**Supplementary Figure 2. The restoration of miR-141 level in BRD7-overexpressing NPC cell models.** qRT-PCR detection confirmed the expression of miR-141 in the miR-141 mimic (miR-141M) or negative control (miR-NC) transfected BRD7-overexpressing NPC cells. U6 serve as an internal control. Control: Vector+miR-NC, BRD7: BRD7+miR-NC. The error bars are presented as the mean ± S.E.M. ***P<0.001. All experiments were performed in triplicate.
